# Substituent effect on inter-ring interaction in paracyclophanes

**DOI:** 10.1007/s11030-019-09926-7

**Published:** 2019-02-19

**Authors:** Irena Majerz, Teresa Dziembowska

**Affiliations:** 1 Faculty of Pharmacy, Wroclaw Medical University, Borowska 211a, 50-556 Wrocław, Poland; 2 Department of Organic Chemistry, Faculty of Chemical Technology, West Pomeranian University of Technology, 70-061 Szczecin, Poland

**Keywords:** [2.2]Paracyclophane, [2.2]Paracyclophane-7,9-dienes, [3.3]Paracyclophanes QTAIM, NCI, AIE, SE

## Abstract

The theoretical calculations, namely multipole-derived charge analysis, quantum theory of atom in molecules, and non-bonding interaction (NCI), were performed for [2.2]paracyclophanes, [2.2]paracyclophane-7,9-dienes, and [3.3]paracyclophanes optimized at B3LYP/6-311++G** level, including dispersion correction. The substituent effect of the electron donor N(Me)_2_ and electron acceptor NO_2_ group and the influence of the length of bridges joining the aromatic ring on aromatic ring interaction energy (AIE) and strain energy were discussed. The local and electrostatic character of the substituent effect in paracyclophanes was shown. The presence of the weak orbital through-space C···C interaction between the [3.3]paracyclophane ring and weak CH···O hydrogen bonds between the substituents in the different rings was shown.

**Electronic supplementary material:**

The online version of this article (10.1007/s11030-019-09926-7) contains supplementary material, which is available to authorized users.

## Introduction

[2.2]Paracyclophanes have attracted interest for more than 20 years, and this interest has not abated yet. Due to their electronic properties and rigid structure, [2.2]paracyclophanes have found an application in manifold branches of chemistry and technology. As an example, let us mention the designing of optoelectronic materials [1, 2], materials for photonics and electronics [3], catalysts in polymer synthesis [4], synthesis of chiro-optically active compounds [5, 6], as well as in biology and medicine [7] or supramolecular chemistry [8]. One of the important challenges is the synthesis of new functionalized [2.2]paracylophanes and the investigation of the substituents’ influence on their chemical, electronic, and structural properties [3].

The crucial problem is to understand the nature of through-space interaction in [2.2]cyclophanes and the substituents’ influence on this interaction. Studies of the isolated cyclophanes permit the investigation of the nature of interactions in the ground state and may be helpful in understanding the role of an interaction in more complex systems. The *π*–*π* stacking interaction between the aromatic rings in [2.2]paracyclophane [9–12] and its complexes [13] was the subject of theoretical investigations, but it has not been fully explained. Close proximity of two aromatic rings of [2.2]cyclophane leads to repulsive as well as attractive interaction between the *π* electrons of both aromatic rings. By analogy with the widely investigated benzene dimers [11, 14–17], the energy of the *π*–*π* interaction between the parcyclophane aromatic rings may be presented as a sum of electrostatic, dispersive, orbital interactions and the Pauli repulsion contribution. For benzene dimers, the dispersion was shown to be the dominant attractive contribution to the binding energy.

Lysenko et al. [10], using the AIM analysis, evidenced that the charge-transfer interaction in [2.2]paracyclophane did not occur. Caramori and Galembeck [9] applied the NBO, MOs, and AIM analysis to investigate [2.2]paracyclophane and *trans* and *cis* [2.2]methacyclophanes. The NBO analysis showed the presence of a significant through-space interaction involving the occupied and unoccupied orbitals (*π*–π*) in [2.2]metacyclophane. It is only for this compound that the AIM method confirmed the existence of the through-space interaction between different metacyclophane rings. The authors [9, 10] have not considered other attractive through-space interactions. Kamya and Muchall [18] studied the structure and inter-ring interactions for several cyclophanes. He assumed that the attractive through-space interaction was controlled by dispersion and electrostatic interaction and, for the shorter distance, the exchange–repulsion dominated. The author showed that the explicit account of the electron correlation effects between the two aromatic rings is crucial for a correct description of the structures of cyclophanes. He named this interaction the “overlap-dispersive,” to differentiate it from the classical dispersion (van der Waals) interactions. Recently, Grimme and Mück-Lichtenfeld [12] calculated the aromatic interaction energies (AIE) and strain energies (SE) for some [2.2]para- and [2.2]metacyclophanes and some other cyclophanes which possessed greater aromatic rings. The AIE presents aromatic interaction energy without taking into account the energy of geometric distortion. The strain energy can be obtained in a cycle of homodesmic reaction, linking the main parts of the molecule.

An influence of the substitution on the structure and nature of *π*–*π* interaction in optimized molecules of cyclophanes was also investigated in a few papers [13, 18–21], but the problem is still far from being elucidated. This effect was more thoroughly studied for benzene dimers [15, 22–26]. Recently, Watt et al. [25] and Wheeler and Bloom [16] reviewed the discussion and controversies concerning the substituent effect on the non-covalent through-space interactions in benzene dimers. Wheeler and Houk [24] proposed a local, direct interaction model. The substituent effect was dominated by electrostatic interaction of the local dipoles associated with the substituent and the electric field of another ring [16, 25]. The mezomeric effect of the substituent on the *π*-electron density was present but was not responsible for substituent effects in benzene dimers. It is noteworthy that the historically first, Hunter and Sanders’, electrostatic model [26] explained the substituent effect by the polarization of the *π*-electron system depending on the electronic properties of the substituent. The analysis of partitioning energy for substituted benzene showed that the electrostatic and dispersive interaction was the most important attractive contribution to the binding energy [15, 16, 24, 25]. Watt et al. [25] found for multi-substituted benzene dimers a fairly good correlation between the calculated energy of the through-space interaction and the sum of the absolute values of the Hammet parameter *σ*_m_ describing the inductive/field effects of the substituent (Σ|*σ*_m_|). The authors showed that the sum of the dispersion, inductive, and exchange contribution to the total binding energy was almost the same for all substituents and the electrostatic energy varied with the substituent according to the Σ|*σ*_m_| values. The results of investigation of the substitution effect in benzene dimers may be helpful in examining this effect in cyclophanes. However, up to now, the results for cyclophanes are rather scant and inconclusive. Frontera et al. investigated the substituent effect on the interaction energy of [2.2] and [3.3]paracyclophane complexes with Na^+^ and Li^+^ cations. The existence of the through-space substituent effect was evidenced. The electron donating NH_2_ or electron-withdrawing CN substituents in one ring increased or reduced the binding energy of the cations with another ring, respectively. Partitioning the total interaction energy into electrostatic, van der Waals, and polarization contributions showed that the substituent effect on the electrostatic contribution is the most important. The QTAIM analysis confirms the presence of through-space interactions. Caramori and Galembeck [19] analyzed the structure and the through-space interaction in tetrafluoro[2.2] para and metacyclophanes. The existence of the through-space *π*–π* for metacyclophane orbital interaction was evidenced by the QTAIM analysis. Galembeck and Caramori [20] studied the influence of F, CN, CO, NH_2_ substitution and protonation effects on the geometry and through-space interactions in a series of [2.2]paracyclophanes. The authors showed the influence of substituents on the dihedral angles of bridges and that this effect did not depend on the electronic properties of the substituents. The presence of numerous through-space unstable interactions in the substituted cyclophanes was suggested. Kamya and Muchall [18] showed the influence of donor–acceptor properties of the substituents on the geometric parameters and the ionization potentials and excitation energies of [*n*.*n*]pracyclophanes. The existence of the charge-transfer process in the substituted cyclophanes was evidenced.

In the previous paper [21], we investigated interactions between the aromatic rings of a series of [2,2]paracyclophanes using QTAIM and NCI analysis in both crystal structure and optimized [2.2]paracyclophanes with the parallel-displaced aromatic rings and additional bridges, and the existence of the through-space orbital interaction was stated. Contrary to expectation, no influence of the substituent effect on these interactions was observed. It is notable that, as mentioned above, no “classical” substituent effect on the stacking interactions in benzene dimers was present. In order to study the substituent effect on the *π*–*π* through-space interaction in the paracyclophane more thoroughly, we have undertaken the calculation of energy of aromatic ring interaction AIE and steric strain SE as well as the calculation of the multipole-derived charges on the aromatic ring carbon atoms in a series of optimized [2.2]paracyclophanes, [2.2]paracyclophane-7,9-dienes, and [3.3]paracyclophanes substituted with the electron-withdrawing NO_2_ and electron-donating N(CH_3_)_2_ substituents.

## Computational details

Optimization of the investigated cyclophanes: [2.2]paracyclophanes, [2.2]paracyclophane-7,9-dienes, and [3.3]paracyclophanes and their derivatives with combinations of NO_2_ and N(CH_3_)_2_ groups was performed at the DFT B3LYP/6-311++G**-DG3 level of calculation including Grimme dispersion [27] using the Gaussian 09 program [28]. The wave functions evaluated for optimized and crystal structure molecules were used as an input to the AIMALL [29] and NCI [30] program. The multipole-derived charge analysis implemented in the ADF program [31, 32] was performed for the molecule optimized with Gaussian without further optimization.

## Results

### Substituent effect on the geometry, AIE, and SE energies for [2.2]cyclophanes, [2.2]cyclophane-1.9-dienes, and [3.3]cyclophanes

The structure of [2.2]paracyclophane was an object of numerous experimental and theoretical studies [10–13, 33, 34]. Recently, Wolf et al. [34] definitely stated that [2.2]paracyclophane below 45 °C crystallizes in a twisted form with an angle of 12.83(4)° and at high temperature in a parallel form. Also the X-ray structure of [2.2]paracyclophane-7,9-diene is known [35], and the heat of formation of this compound was studied. The structure of [3.3]paracyclophane was determined by experimental [36, 37] and theoretical methods [33].

**Scheme 1:**
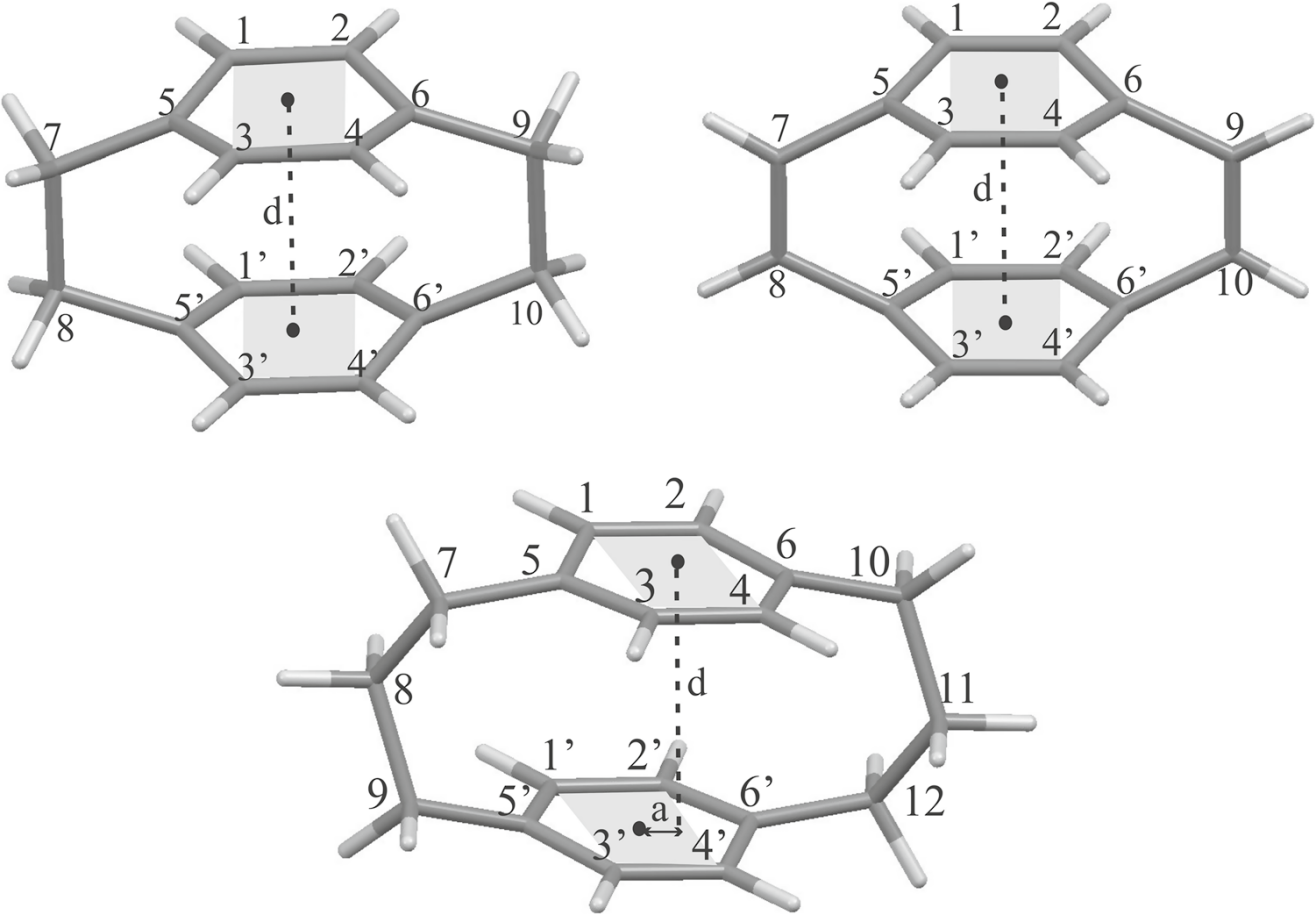
Atom numbering for the investigated cyclophanes. *d* Distance between the ring planes, *a* ring displacement; the planes marked in gray are considered to represent the angle between the ring planes (*α*)

To characterize the conformation of the investigated paracyclophanes, we have used the following parameters: the distance between the benzene rings, the displacement of one aromatic ring relating to another (d in Scheme 1), and the twist of one ring in relation to another ring (the angle between the gray planes in Scheme 1) [21].

The values of the distance between the rings (*d*), ring displacement (*a*), and the angle between the aromatic ring planes (*α*) for paracyclophanes are presented in Table 1.

**Table 1:** Geometrical parameters for cyclophanes according to Scheme 1

	[2.2]Cyclophane			[2.2]Cyclophane-1.9-diene			[3.3]Cyclophane		
	*d* [Å]	*a* [Å]	*α* [°]	*d* [Å]	*a* [Å]	*α* [°]	*d* [Å]	*a* [Å]	*α* [°]
Cyclophane	3.1527	0.0435	0.000	3.1660	0.0000	0.001	3.3055	0.1060	2.586
1-Nitro	3.1238	0.0353	0.851	3.1492	0.0905	1.500	3.3055	0.1060	2.586
1-Dimethylamino	3.1271	0.1522	0.433	3.4255	0.5583	10.632	3.3372	0.1734	0.760
1-Dimethylamino	3.1607	0.4186	5.238	3.2047	0.1410	5.162	3.4303	0.3529	11.700
1′-Dimethylamino									
1-Dimethylamino	3.1549	0.1005	5.296	3.1505	0.0561	0.009	3.3465	0.5934	0.651
4′-Dimethylamino									
1-Dimethylamino-2-nitro	3.1426	0.1525	0.802	3.1281	0.4291	0.359	3.2459	0.7347	2.190
3′-Dimethylamino-4′-nitro									
1,2-Dimethylamino	3.1489	0.1177	2.745	3.1111	0.5446	0.790	3.1805	0.9862	7.241
3′,4′-Nitro									
1-Nitro	3.1312	0.0936	1.2070	3.1600	0.1509	2.393	3.2994	0.4714	3.342
1′-Nitro									
1-Nitro-4-dimethylamino	3.1445	0.1484	1.4070	3.1454	0.1738	0.003	3.3266	0.3236	4.032
1′-Dimethylamino-4′-nitro									
1-Dimethylamino	3.1473	0.1739	5.5240	3.1427	0.0793	0.535	3.3320	0.1125	4.891
4′-Nitro									
1-Dimethylamino	3.1373	0.0937	1.1270	3.1352	0.2443	1.453	3.3128	0.5473	6.272
1′-Nitro									
1-Dimethylamino-4-nitro	3.162	0.3911	3.1500	3.1978	0.1842	1.847	3.3388	0.2798	3.448
1′-Dimethylamino-4′-nitro									

#### Substituted [2.2]paracyclophanes

The optimized geometry of [2,2]paracyclophane indicates a parallel structure with a very short interplanar distance of 3.153 Å, much shorter than the sum of van der Waals radii which, for the carbon atom, is 3.4 Å. The introduction of a substituent into the benzene ring of the cyclophane leads to small changes of the interplanar distance (d), ring displacement (a), and the angle between the ring planes (*α*). This indicates the presence of a strained, close-to-parallel structure of [2.2]cyclophanes and suggests a strong repulsion between the *π*-electrons of both aromatic rings. The energy of aromatic interaction (AIE) [12] has been computed as the difference between the energy of cyclophane molecules where the bridges were removed and substituted by the H atom of exactly the same geometry. The AIE value reveals a strong repulsion interaction between the benzene rings in [2.2]paracyclophane. The value of AIE for [2.2]paracyclophane is + 12.863 kcal/mol, close to that determined by Grimme (14.3 kcal/mol) [12]. The positive AIE values obtained for the majority of the analyzed substituted cyclophanes, with the exception of 1′,4-bis(dimethylamino)-1,4′-dinitro[2.2]paracyclophane, show the dominance of the repulsive interactions between the aromatic rings. The AIE values indicate that the introduction of substituents to the aromatic rings leads to a decrease in the repulsive character of the interaction between the aromatic rings, independently of the electron-donating or electron-withdrawing properties of the substituent. The unexpected negative AIE values observed for cyclophane, where two N(CH_3_)_2_ substituents in one ring are situated over two NO_2_ substituents in another ring, may be explained by the possibility of some local interactions between the substituents. The steric strain energy (SE) has been calculated following Grimme and Mück-Lichtenfeld [12] and Bachrach [19], employing the homodesmic reaction (Table 2). For [2.2]paracyclophane, this value is 44.01 kcal/mol and increases after the substituent introduction.

**Table 2:** Aromatic interaction and steric energies for investigated cyclophanes

	[2.2]Cyclophane		[2.2]Cyclophane-1.9-diene		[3.3]Cyclophane	
	AIE [kcal/mol]	SE [kcal/mol]	AIE [kcal/mol]	SE [kcal/mol]	AIE [kcal/mol]	SE [kcal/mol]
Cyclophane	12.86	44.01	13.43	53.09	3.29	9.84
1-Nitro	11.09	52.20	13.33	47.85	− 1.07	6.96
1-Dimethylamino	11.98	55.50	3.32	124.73	− 0.19	7.95
1-Dimethylamino	− 2.72	73.15	10.61	47.40	− 0.23	9.52
1′-Dimethylamino						
1-Dimethylamino	8.98	73.15	9.35	79.55	− 1.62	6.13
4′-Dimethylamino						
1-Dimethylamino-2-nitro	4.22	77.44	8.64	43.53	− 4.78	4.54
3′-Dimethylamino-4′-nitro						
1,2-Dimethylamino	5.58	80.63	5.71	42.05	− 5.38	4.61
3′,4′-Nitro						
1-Nitro	11.19	64.67	14.67	46.77	− 1.78	8.34
1′-Nitro						
1-Nitro-4-dimethylamino	− 2.89	66.72	− 4.42	24.89	− 11.05	− 9.00
1′-Dimethylamino-4′-nitro						
1-Dimethylamino	8.65	69.48	10.75	43.35	− 3.03	5.53
4′-Nitro						
1-Dimethylamino	6.97	63.71	5.91	40.66	− 3.94	5.83
1′-Nitro						
1-Dimethylamino-4-nitro	5.31	73.75	9.05	37.85	− 5.32	7.33
1′-Dimethylamino-4′-nitro						

#### [2.2]Paracyclophane-7,9-diene

Geometric parameters for [2.2]paracyclophane-1,9-diene are given in Table 1. Contrary to expectation, introduction of the double bond to the bridge does not cause shortening of the inter-ring distance compared to those found for [2.2]paracyclophane. Similarly, the ring displacement between the aromatic rings and the angle between them are close to the parameters for [2.2]paracyclophane. The AIE value indicates the strong strain structure and dominance of steric repulsion in the through-space interaction. The AIE of 13.434 kcal/mol is slightly larger than for [2.2]paracyclophane and decreases with the introduction of the substituents. Only for the derivative with 1′,4-bis(dimethylamino)-1,4′-dinitro-substituent does the negative AIE value of − 4.419 kcal/mol indicates a dominance of attractive interactions. The strain energy (SE) for [2.2]paracyclophane-7,9-diene shows the less strained structure compared to [2.2]paracyclophane. This fact indicates an important contribution of steric repulsion caused by the eclipsing interactions between the CH_2_ groups in [2.2]paracyclophanes.

#### Substituted [3.3]paracyclophanes

The geometric parameters for [3.3]paracyclophanes show that the elongation of aliphatic bridges causes an important increase in the inter-ring distance in comparison with [2.2]paracyclophane. The interplanar shift (d) and interplane (*α*) angle indicate the parallel–displaced structure with a small angle between the aromatic planes. An increase in the interplanar distance from 3.15 Å for [2.2]paracyclophane to 3.31 Å for [3.3]paracyclophane reduction leads to a decrease in the AIE from 12.86 to 3.153 kcal/mol. For substituted [3.3]paracyclophanes, the negative AIE values, indicating the dominance of the attractive interactions, increase with the number of substituents. An increase in the inter-ring distance in comparison with [2.2]cyclophanes results in a decrease in the destabilizing Pauli repulsion energy. The values of the strain energy (SE) indicate the smallest steric strain in this series of cyclophanes under study.

#### Discussion of the NO_2_ and NMe_2_ substituent effect on AIE and SE energy for [2.2]cyclophanes, [2.2]cyclophane-1.9-dienes, and [3.3]cyclophanes

Comparison of the AIE and SE values for substituted [2.2]paracyclophane, [2.2]paracyclophane-7,9-diene, and [3.3]paracyclophane shows that the introduction of both electron donor NMe_2_ and electron acceptor NO_2_ groups increases the attractive interaction between the aromatic rings. This result shows that the substituent effect in paracyclophanes is predominated by the electrostatic interactions.

In [2.2] paracyclophanes, the repulsive interaction between the aromatic rings prevails on the attractive one and decreases the positive values of AIE. The very short inter-ring distances in these compounds mean that the *π*-orbitals of the different rings may be overlapped. The destabilizing interaction between the occupied orbitals (Pauli repulsion energy) seems to be mainly responsible for positive AIE values. As was shown by Grimme [11], in [2.2]paracyclophane, the LUMO orbital has a considerable bonding character that leads to decreasing Pauli repulsion and attractive “overlap-dispersive” interaction.

The steric strain and the aromatic interaction energy increases with a growing number of substituents. The lack of correlation between the SE and AIE for substituted [2.2] paracyclophanes is the result of the contribution of the strain energy of the alkyl bridges.

Correlation of the SE values with the hardness parameter (*η*) [*η* = *E*_LUMO_ − *E*_HOMO_ [38] (Fig. S1) shows that the strain energy destabilizes the cyclophane molecule.

The results obtained for paracyclophanes show that the attractive intra-space interactions between the aromatic rings increase with the number of substituents, independently of their electronic character. Watt et al. [25] observed a similar effect for benzene dimers and found a correlation between the binding energy and the sum of the absolute value of the field/inductive Hammet constant Σ|*σ*_m_| [39]. The authors stated [25] that this parameter contains some information about the substituent’s effect on electrostatic and dispersion contribution to the binding energy. Following Watt et al. [25], we have examined the relationships between the AIE values and the sum of absolute values of inductive Hammett parameters Σ|*σ*_m_|. For [2.2]paracyclophane, [2.2]paracyclophane-7,9-diene, where the repulsive interactions are dominant, no relationships are found. For [3.3]paracyclophane (Fig. S3), the tendency of an increase in AIE with increasing Σ|*σ*_m_| [40] (Fig. S2) permits us to suggest that both electrostatic and dispersion effects contribute to the energy of an interaction between the aromatic rings in [3.3]paracyclophanes. It is to be noted that only a small quantity of data have been available and the presence of direct interaction between substituents in the multi-substituent derivatives influences the AIE values, as has been suggested above. As was shown for benzene dimers [25], an increase in the surface area in multi-substituted benzenes leads to an increase in the contribution of dispersion in the binding energy, but this effect may be compensated by increasing repulsive interaction. The tendency of an increase in the AIE values with the sum of the molar refractivity substituent constant ΣMr [40] (Fig S3), which characterizes the dispersion/polarizability interaction, confirms the importance of the contribution of the dispersive interaction for the AIE values.

### The multipole-derived charges on the carbon atoms in the aromatic rings of substituted [2.2]paracyclophane, [2.2]paracyclophane-7,9-diene, and [3.3]paracyclophane

The multipole-derived charges on the carbon atoms presented in Table 1 show that an introduction of electron donor N(CH_3_)_2_ and electron acceptor NO_2_ groups to the aromatic ring causes a decrease in the negative charges on the substituted carbon atom in the aromatic ring of all the investigated cyclophanes, and this effect is more significant for the N(CH_3_)_2_ than for the NO_2_ group. Only small changes are observed for unsubstituted carbon atoms in the rings and carbon atoms of the aliphatic bridges in [2,2]paracyclophanes and [3.3]paracyclophane. In [2.2]cyclophane-1.9-diene, the multipole-derived charge on 5,5′ C atoms and atoms of the bridges are sensitive to the substituent effects. A similar effect is observed in mono- and multi-substituted cyclophane derivatives. The observation that the substituents lead to a decrease in the negative charge on the carbon atom of aromatic rings connected with the substituent, independently of their electron-withdrawing or electron-donating character, confirms the local, electrostatic origin of the substituent effect for the investigated paracyclophanes.

### QTAIM analysis of substituted [2.2]paracyclophane, [2.2]paracyclophane-7,9-diene, and [3.3]paracyclophane

In order to obtain information concerning the presence of the through-space orbital interactions between the C atoms of the aromatic rings, we have performed the Quantum Theory Atom In Molecule (AIM, QTAIM) analysis [41–43]. The QTAIM analysis is a very efficient tool in the investigation of closed-shell interactions, like conventional hydrogen bonds, H···H interactions, and various weakly bonded complexes [42–47]. The presence of the bond path between the given atoms with the electron density at the bond-critical point (BCP) *ρ*(*r*) < 0.1 a.u. and positive Laplacian of the electron density are typical for closed-shell interactions. The small ellipticity of electron density (*ε*) at the BCP and linearity of the bond are an indication of the stable interaction [48].

The QTAIM analysis has been previously applied in the studies of [2.2]paracyclophane [10, 11], [2.2]*meta*cyclophane [9], and [*n*,*n*]paracyclophane complexes with cations [13]. In the previous paper [21], we applied the QTAIM analysis to investigate the through-space interactions in a series of unsubstituted and substituted [2.2]paracyclophanes. The presence of a C···C bond path between the C atoms belonging to different rings in the [2.2]paracyclophane molecule and the charge density at the BCP *ρ*(*r*) > 0.0125 a.u. were taken as evidence for the presence of through-space orbital interactions between the C atoms [21]. For [2.2] paracyclophane and [2.2]paracyclophane-7,9-diene and their derivatives investigated in this work, no bond path between the C···C atoms in the different rings has been detected. For [3.3]paracyclophane, two bond paths with the *ρ*(*r*) of 0.0076 a.u. have been observed, which suggests the existence of a weak orbital interaction of the C atoms in different rings. As can be seen, the decreased strong repulsion interaction between the aromatic rings by increasing through-bond distance permits the appearance of very weak orbital interactions.

For the three groups of substituted paracyclophanes investigated in this work, the bond paths with the *ρ*(*r*) values from 0.0076 to 0.0083 a.u. have been observed, but only some of them are indicators of the presence of a stable orbital interaction between the C atoms in different rings. No influence of the substituents on the charge density at the BCP, and hence the energy of the C···C interaction, has been observed. QTAIM analysis evidences the existence of numerous non-covalent interactions between the substituents. The interactions between the O atom of the NO_2_ group and the H atom of the N(CH_3_)_2_ group, present in the same or different ring in a vicinal position and situated one above another in different rings, have been observed. These interactions may be assigned to the CH···O hydrogen bond. Also the O···H interactions between the nitro group and the H atom of the aromatic ring as well as the H···H interactions between the H atoms of the methyl group and H atoms of the bridges and the aromatic rings are shown. The CH···O hydrogen bonds between the nitro and dimethylamino group in different rings are worth noticing, particularly for fourthly substituted cyclophanes with 1′,4-bis(dimethylamino) and 1,4′-dinitro groups. For 1′,4-bis(dimethylamino)-1,4′dinitro [2.2]paracyclophane, an H···O bond path with *ρ*(*r*) of 0.0052 a.u. has been observed. For the [3.3]paracyclophane analog of this compound, two H···O bond paths with a *ρ*(*r*) value of 0.0114 and 0.0086 a.u. and one N···O bond path (with *ε* > 1) have been found. For the same derivative of [2.2]paracyclophane-7,9-diene, the H···O bond paths with *ρ*(*r*) equal to 0.0113, 0.0071, and 0.0071 a.u. and two N···N bond paths (with *ε* > 1) have been shown. The presence of the stable CH···O interactions between the substituents in the different rings, apart from the through-space stacking interactions between the aromatic rings, contributes to the total AIE value. This may explain the exceptional negative AIE values: − 2.893, − 4.419, and − 11.054 kcal/mol for [2.2]paracyclophane and [2.2]paracyclophan-7,9-diene, and [3.3]paracyclophane, respectively. The above results have shown that in the substituted paracyclophanes the AIE value is not only a measure of the through-bond interaction between the aromatic rings, but has some contribution resulting from the direct through-space interaction between the substituents, which explains the scattering of the points in the AIE versus Σ|*σ*_m_| plot (Fig S2).

We have applied the QTAIM analysis to answer the question of whether the atoms of the bridges joining the aromatic rings can participate in the interactions with the rest of the molecule. The QTAIM plots for the structures in which the bridges linking the aromatic rings have been replaced by the H atoms show that interactions in those molecules are different. The most significant difference in the QTAIM plots of the cyclophanes and their analogs in which the CH=CH and CH_2_CH_2_ bridges have been replaced by the H atoms is the appearance of new bond paths joining the H atoms added in different rings. At the same time, the bond paths joining the atoms of the substituents with the H atoms of the bridges have disappeared. The values of *ρ*(*r*) for the through-space C···C interaction remain the same, but the increasing ellipticity of the electron density at BCP and nonlinearity of the C···C bond path suggests an increase in instability of these interactions. The results show that a removal of the cyclophane bridges causes some changes in the very weak orbital interactions between the aromatic rings.

### NCI analysis of substituted [2.2]paracyclophane, [2.2]paracyclophane-1,9-diene, and [3.3]paracyclophane

To obtain information concerning the dispersive interactions in investigated cyclophanes, the non-covalent interaction (NCI) approach has been applied [17, 49]. This method, which is an efficient tool to analyze and visualize weak non-covalent interactions, is based on the plot of the reduced density gradient versus the electron density multiplied by the sign of the second Hessian eigenvalue (λ_2_). Multiplication of electron density by the sign of the second Hessian eigenvalue differentiates the repulsive (signλ_2_)*ρ*(*r*) > 0 and attractive (signλ_2_) *ρ*(*r*) < 0 interactions. Typical dispersion interactions usually appear as spikes at very low density values (*ρ*(*r*) < 0.01 a.u.), whereas stronger interactions, as hydrogen bonds, appear at higher density values (0.01 < 1<0.05 a.u.) of the NCI plot [46, 47]. The second possibility of presentation of the non-covalent interactions in the frame of the NCI method is the visualization of electron-density-gradient isosurfaces in real space for the molecule and typical colors present a particular type of interaction: blue for attractive, red for repulsive, and green for intermediate interaction. NCI analysis for [2.2]paracyclophanes was performed previously [21].

**Fig. 1:**
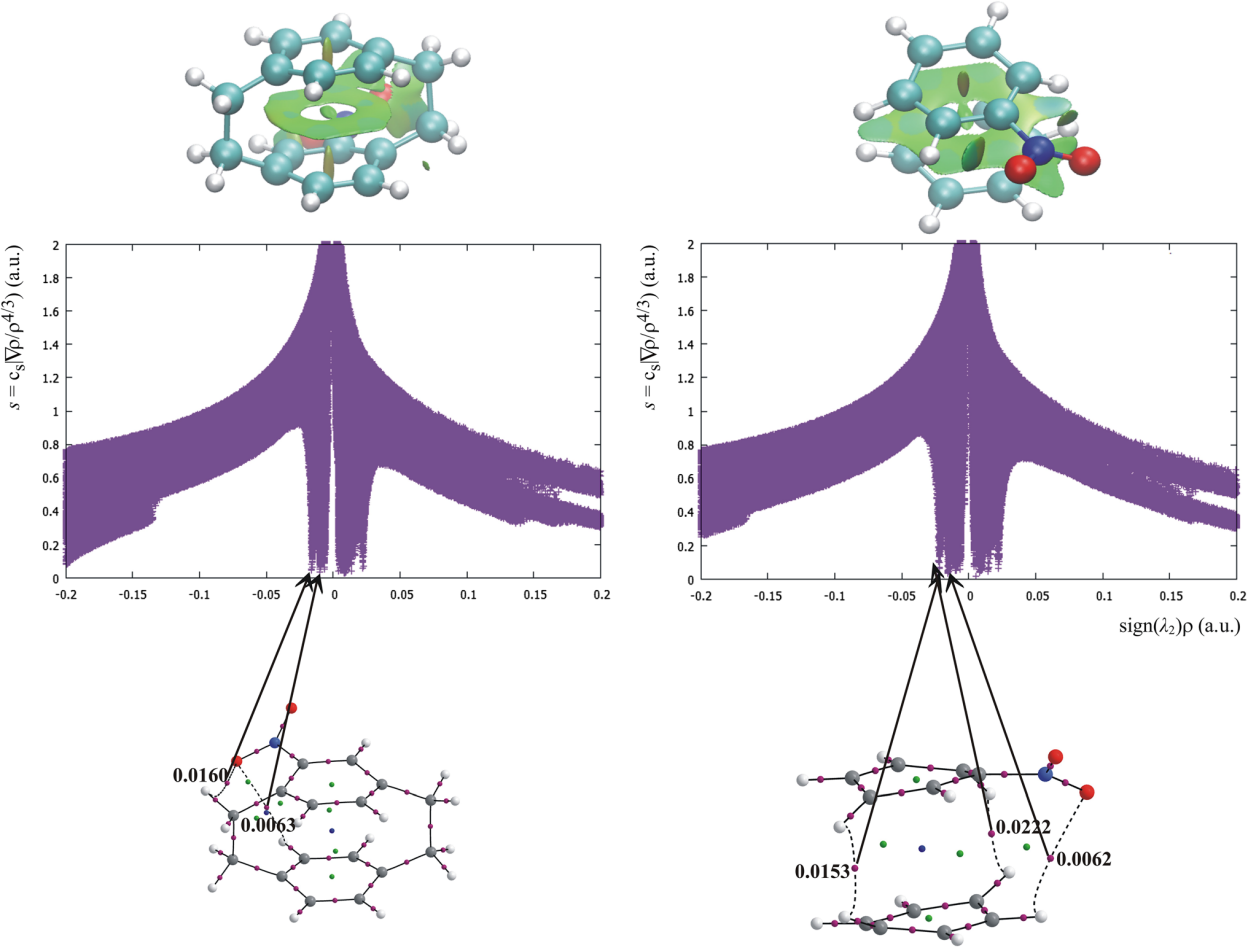
NCI surface and NCI plot for 1-nitro[2.2]paracyclophane (left) and its analog without the aliphatic bridges (right) compared with the AIM diagram

The NCI analysis for the investigated cyclophanes evidenced the existence of numerous attractive and repulsive weak non-bonding interactions, presented by positive and negative *ρ*(*r*) values for the spikes in the NCI plot. For all cyclophanes, repulsive interactions dominate over the attractive ones. For substituted, and particularly for the multi-substituted paracyclophanes, the number of spikes corresponding to both attractive and the repulsive interaction increases. The non-bonding interactions have been visualized by green gradient surfaces. The gradient surface shows that the weak non-bonding interactions are present not only between the aromatic rings, but also between some of the substituents in a vicinal position or closely situated in both the rings and also between the substituents in the bridges and the aromatic rings (Fig. 1).

## Conclusions

1. The aromatic interaction energy (AIE) as well as the steric energy (SE) for the investigated cyclophanes is influenced by the structure and length of the bridges joining the aromatic rings. For [2.2] paracyclophanes, the positive AIE values indicate the dominance of repulsive interactions, while for substituted [3.3]paracyclophanes, the negative AIE values indicate the dominance of attractive interaction.
2. The AIE values for paracyclophanes under study show that the introduction of substituents into the benzene rings, independently of their electronic character, increases the attractive interaction between the aromatic rings. This result shows that the electrostatic interactions play a dominant role in the substituent effect on the attractive through-space interaction in paracyclophanes.
3. An analysis of the multipole-derived charges on the carbon atoms in aromatic rings of paracyclophanes under study suggests that the substituents’ effect on through-space interactions between the aromatic rings is controlled by local, electrostatic interactions.
4. The QTAIM analysis evidences the existence of the weak orbital through-space C···C interaction between different rings for [3.3]paracyclophane and in some substituted derivatives. For substituted [2.2] and [3.3]paracyclophanes, the presence of numerous non-covalent interactions between the atoms of the NO_2_ and NMe_2_ groups and the H atoms of the aliphatic bridges and aromatic ring was stated.
5. Formation of the through-space CH···O hydrogen bonds between NO_2_ and protons from the N(CH_3_)_2_ group or other aliphatic or aromatic protons stabilizes the stacking interaction between the aromatic rings in the paracyclophanes under study.
6. The NCI plots for the investigated compounds evidence the presence of dispersive and other non-bonding attractive and repulsive interactions between the aromatic rings. The gradient surfaces confirmed the existence of weak interactions between the aromatic rings and the substituents. The comparison of the gradient surface for the structure where the bridges are replaced by the H atoms with the parent compound shows differences in the non-covalent interactions.

## Electronic supplementary material

Below is the link to the electronic supplementary material.

Supplementary material 1 (DOCX 161 kb)

## References

[1] Zyss Y, Ledoux I, Volkov S, Chernyak V, Mukamel S, Bartholomew GP, Bazan GC (2000). Through-space charge transfer and nonlinear optical properties of substituted paracyclophane. J Am Chem Soc.

[2] Bartholomew GP, Bazan GC (2001). Bichromophoric paracyclophanes: models for interchromophore delocalization. Acc Chem Res.

[3] Marrocchi A, Tomasi I, Vaccaro L (2012). Organic small molecules for photonics and electronics from the [2.2]paracyclophane scaffold. Isr J Chem.

[4] Camacho DH, Salo EV, Ziller JW, Guan Z (2004). Cyclophane-based highly active late-transition-metal catalysts for ethylene polymerization. Angew Chem Int Ed.

[5] Gon M, Morisaki Y, Chujo Y (2015). Optically active cyclic compounds based on planar chiral [2.2]paracyclophane: extension of the conjugated systems and chiroptical properties. J Mater Chem C.

[6] Sergeeva EV, Rozenberg VI, Antonov DYu, Vorontsov EV, Starikova ZA, Fedyanin IV, Hopf H (2005). Novel multichiral diols and diamines by highly stereoselective pinacol coupling of planar chiral [2.2]paracyclophane derivatives. Chem Eur J.

[7] Lahann J, Hocker H, Langer RD (2001). Synthesis of amino[2.2]paracyclophanes—beneficial monomers for bioactive coating of medical implant materials. Angew Chem Int Ed.

[8] Frišcic T, MacGillivray LR (2005). Cyclophanes and ladderanes: molecular targets for supramolecular chemists. Supramol Chem.

[9] Caramori GF, Galembeck SE (2007). Computational study about through-bond and through-space interactions in [2.2] cyclophanes. J Phys Chem A.

[10] Lyssenko KA, Antypin MYu, Antonov DYu (2003). The transannular interaction in [2.2]paracyclophane: repulsive or attractive?. ChemPhysChem.

[11] Grimme S (2004). On the importance of electron correlation effects for the π–π interactions in cyclophanes. Chem Eur J.

[12] Grimme S, Mück-Lichtenfeld C (2012). Accurate computation of structures and strain energies of cyclophanes with modern DFT methods. Accurate computation of structures and strain energies of cyclophanes with modern DFT methods. Isr J Chem.

[13] Frontera A, Quinonero D, Garau C, Costa A, Ballester P, Deya PM (2006). Ab initio study of [n.n]paracyclophane (n = 2, 3) complexes with cations: unprecedented through-space Substituent effects. J Phys Chem A.

[14] Grimme S (2008). Do special noncovalent π–π stacking interactions really exist?. Angew Chem Int Ed.

[15] Sinnokrot MO, Sherrill CD (2004). Substituent effects in π–π interactions: sandwich and T-shaped configurations. J Am Chem Soc.

[16] Wheeler SE, Bloom JWG (2014). Toward a more complete understanding of noncovalent interactions involving aromatic rings. J Phys Chem A.

[17] Johnson ER, Keinan S, Mori-Sanchez P, Contreras-Garcia J, Cohen AJ, Yang W (2010). Revealing noncovalent interactions. J Am Chem Soc.

[18] Kamya PRN, Muchall HM (2008). New insights into the use of (TD-)DFT for geometries and electronic structures of constrained π-stacked systems: [*n*.*n*]paracyclophanes. J Phys Chem A.

[19] Caramori GF, Galembeck SE (2008). A computational study of tetrafluoro-[2.2] cyclophanes. J Phys Chem A.

[20] Galembeck SE, Caramori GF (2006) Effect of Substituents and Protonation on the Electronic Structure of [2.2] Paracyclophane. IUPAC International Conference on Physical Organic Chemistry (ICPOC-18), Warsaw, Poland, 20–25 August

[21] Majerz I, Dziembowska T (2016). Aromaticity and through-space interaction between aromatic rings in [2.2] paracyclophanes. J Phys Chem A.

[22] Hunter CA, Lawson KR, Perkins J, Urch CJ (2001). Aromatic interactions. J Chem Soc Perkin Trans.

[23] Sherrill CD, Sumpter BG, Sinnokrot MO, Marshall MS, Hohenstein EG, Walker RC, Gould IR (2009). Assessment of standard force field models against high-quality ab initio potential curves for prototypes of π–π, CH/π, and SH/π interactions. J Comput Chem.

[24] Wheeler SE, Houk KN (2008). Substituent effects in the benzene dimer are due to direct interactions of the substituents with the unsubstituted benzene. J Am Chem Soc.

[25] Watt M, Hardebeck LKE, Kirkpatrick CC, Lewis M (2011). Face-to-face arene–arene binding energies: dominated by dispersion but predicted by electrostatic and dispersion/polarizability substituent constants. J Am Chem Soc.

[26] Hunter CA, Sanders JKM (1990). The nature of. pi.-. pi. interactions. J Am Chem Soc.

[27] Grimme S, Antony J, Ehrlich S, Krieg H (2010). A consistent and accurate ab initio parametrization of density functional dispersion correction (DFT-D) for the 94 elements H-Pu. J Chem Phys.

[28] Gaussian 09 (2010) Revision A.9, Gaussian, Inc., Pittsburgh

[29] Keith TA (2014) TK Gristmill software, Overland Park KS, USA. (aim.tkgristmill.com)

[30] http://www.chem.duke.edu/yang/software.htm

[31] te Velde G, Bickelhaupt FM, van Gisbergen SJA, Fonseca Guerra C, Baerends EJ, Snijders JG, Ziegler T (2001). Chemistry with ADF. J Comput Chem.

[32] Fonseca Guerra C, Snijders JG, te Velde G, Baerends EJ (1998) Towards an order-N DFT method. Theor Chem Acc 99:391–403. 10.1007/s002149800m26; ADF2009.01, SCM, Theoretical Chemistry, Vrije Universiteit, Amsterdam, The Netherlands. http://www.scm.com

[33] Bachrach SM (2011). DFT study of [2.2]-,[3.3]-, and [4.4] paracyclophanes: strain energy, conformations, and rotational barriers. J Phys Chem A.

[34] Wolf H, Leusser D, Jørgensen MRV, Herbst-Irmer R, Chen Y-S, Scheidt EW, Scherer WB, Iversen B, Stalke D (2014). Phase transition of [2, 2]-paracyclophane—an end to an apparently endless story. Chem Eur J.

[35] Meijere A, Kozhushkov SI, Rauch K, Schill H, Verevkin SP, Kümmerlin M, Beckhaus H-D, Rüchardt C, Yufit DS (2003). Heats of formation of [2.2] paracyclophane-1-ene and [2.2] paracyclophane-1, 9-diene—an experimental study. J Am Chem Soc.

[36] Ganzel PK, Trueblood KN (1965). The crystal and molecular structure of [3.3] paracyclophane. Acta Cryst B.

[37] Swart M, Van Duijnen PT, Snijders JG (2001). A charge analysis derived from an atomic multipole expansion. J Comput Chem.

[38] Elango M, Parthasarathi R, Subramanian V (2007). Chemical reactivity patterns of [n]paracyclophanes. J Mol Struct Theochem.

[39] Hansch C, Leo A, Taft RW (1991). A survey of Hammett substituent constants and resonance and field parameters. Chem Rev.

[40] http://www.wiredchemist.com

[41] Bader RFW (1990). Atoms in molecules: a quantum theory.

[42] Bone RGA, Bader RFW (1996). Identifying and analyzing intermolecular bonding interactions in van der Waals molecules. J Phys Chem.

[43] Espinosa E, Alkorta I, Rozas I, Elguero J, Molins E (2001). About the evaluation of the local kinetic, potential and total energy densities in closed-shell interactions. Chem Phys Lett.

[44] Wojtulewski S, Grabowski SJ (2003). Ab initio and AIM studies on intramolecular dihydrogen bonds. J Mol Struct.

[45] Hobza P, Havlas Z (2000). Blue-shifting hydrogen bonds. Chem Rev.

[46] Grabowski SJ, Sokalski WA, Leszczynski J (2004). Nature of X–H^+δ^···^−δ^H–Y dihydrogen bonds and X–H···σ interactions. J Phys Chem A.

[47] Lipkowski P, Grabowski SJ, Robinson TL, Leszczynski J (2004). Properties of the C–H···H dihydrogen bond: an ab initio and topological analysis. J Phys Chem A.

[48] Popelier PLA (1998). Characterization of a dihydrogen bond on the basis of the electron density. J Phys Chem A.

[49] Alonso M, Woller T, Martin-Martinez FJ, Contreras-Garcia J, Geerlings P, De Proft F (2014). Understanding the fundamental role of π/π, σ/σ, and σ/π dispersion interactions in shaping carbon-based materials. Chem Eur J.

