# Supplementary material for: Substituent effect on inter-ring interaction in paracyclophanes
Source: Mol Divers. 2019 Feb 19;24(1):11–20. doi: 10.1007/s11030-019-09926-7 (PMC7033074; doi:10.1007/s11030-019-09926-7)
Supplement: Supplementary file 1 — Supplementary material 1 (DOCX 161 kb) [file 11030_2019_9926_MOESM1_ESM.docx]

Table S1. Multipole derived charges (in a.u.) for carbon atoms of the investigated cyclophane derivatives.

Atom numbering according to Scheme 1.

| [2.2]paracyclophane | 1 | 2 | 3 | 4 | 5 | 6 | 7 | 8 |
| --- | --- | --- | --- | --- | --- | --- | --- | --- |
|  | 1’ | 2’ | 3’ | 4’ | 5' | 6' | 9 | 10 |
| [2.2]paracyclophane | -0.247 | -0.247 | -0.247 | -0.247 | -0.095 | -0.095 | 0.262 | 0.262 |
| 1-nitro | -0.031 | -0.236 | -0.222 | -0.201 | -0.014 | -0.136 | 0.220 | 0.215 |
|  | -0.217 | -0.218 | -0.237 | -0.205 | -0.116 | -0.068 | 0.256 | 0.250 |
| 1-dimethylamino | 0.098 | -0.249 | -0.282 | -0.251 | -0.073 | -0.251 | 0.228 | 0.212 |
|  | -0.193 | -0.225 | -0.229 | -0.233 | -0.020 | -0.140 | 0.255 | 0.258 |
| 1-dimethylamino | 0.099 | -0.23 | -0.233 | -0.284 | 0.012 | -0.132 | 0.192 | 0.213 |
| 1’-dimethylamino | 0.097 | -0.213 | -0.282 | -0.275 | -0.061 | -0.043 | 0.244 | 0.247 |
| 1-dimethylamino | 0.165 | -0.136 | -0.199 | -0.248 | -0.048 | -0.133 | 0.214 | 0.227 |
| 4’-dimethylamino | -0.223 | -0.205 | -0.273 | 0.091 | -0.085 | -0.020 | 0.227 | 0.231 |
| 1-dimethylamino-2-nitro | 0.036 | -0.028 | -0.203 | -0.257 | 0.010 | -0.087 | 0.203 | 0.204 |
| 3’-dimethylamino-4’-nitro | -0.212 | -0.228 | 0.055 | -0.017 | -0.051 | -0.033 | 0.216 | 0.212 |
| 1,2- dimethylamino | 0.061 | 0.088 | -0.244 | -0.264 | 0.011 | -0.047 | 0.196 | 0.207 |
| 3’,4’-nitro | -0.175 | -0.173 | 0.005 | -0.066 | -0.072 | -0.009 | 0.208 | 0.216 |
| 1-nitro | -0.029 | -0.18 | -0.241 | -0.201 | -0.081 | -0.061 | 0.212 | 0.210 |
| 1’-nitro | -0.018 | -0.206 | -0.214 | -0.217 | -0.026 | -0.125 | 0.239 | 0.238 |
| 1-nitro-4-dimethylamino | -0.043 | -0.218 | -0.277 | 0.070 | -0.036 | -0.014 | 0.207 | 0.207 |
| 1’-dimethylamino-4’-nitro | 0.075 | -0.252 | -0.202 | -0.056 | -0.028 | -0.066 | 0.213 | 0.210 |
| 1-dimethylamino | 0.164 | -0.130 | -0.218 | -0.261 | -0.052 | -0.146 | 0.209 | 0.223 |
| 4’-nitro | -0.156 | -0.180 | -0.224 | -0.044 | -0.093 | -0.028 | 0.214 | 0.229 |
| 1-dimethylamino | 0.078 | 0.026 | -0.248 | -0.272 | 0.002 | -0.127 | 0.203 | 0.207 |
| 1’-nitro | 0.001 | -0.176 | -0.254 | -0.182 | -0.063 | -0.043 | 0.239 | 0.238 |
| 1-dimethylamino-4-nitro | -0.216 | -0.06 | 0.067 | -0.248 | -0.017 | -0.080 | 0.205 | 0.195 |
| 1’-dimethylamino-4’-nitro | 0.102 | -0.242 | -0.236 | -0.038 | -0.057 | -0.017 | 0.225 | 0.227 |

| [2.2]paracyclophane-7,9-diene | 1 | 2 | 3 | 4 | 5 | 6 | 7 | 8 |
| --- | --- | --- | --- | --- | --- | --- | --- | --- |
|  | 1’ | 2’ | 3’ | 4’ | 5' | 6' | 9 | 10 |
| [2.2]paracyclophane-7,9-diene | -0.250 | -0.250 | -0.237 | -0.237 | 0.074 | 0.074 | -0.067 | -0.066 |
|  | -0.237 | -0.237 | -0.205 | -0.250 | 0.074 | 0.074 | -0.066 | -0.067 |
| 1-nitro | -0.096 | -0.199 | -0.251 | -0.177 | 0.038 | 0.105 | 0.021 | 0.012 |
|  | -0.200 | -0.256 | -0.221 | -0.240 | 0.102 | 0.007 | 0.006 | 0.050 |
| 1-dimethylamino | 0.032 | -0.264 | -0.276 | -0.310 | 0.107 | 0.079 | -0.092 | -0.221 |
|  | -0.239 | -0.179 | -0.271 | -0.143 | -0.008 | 0.105 | -0.06 | 0.057 |
| 1-dimethylamino | 0.102 | -0.242 | -0.275 | -0.279 | -0.022 | 0.116 | 0.026 | -0.006 |
| 1’-dimethylamino | 0.111 | -0.250 | -0.220 | -0.310 | 0.057 | 0.019 | 0.036 | 0.036 |
| 1-dimethylamino | 0.094 | -0.278 | -0.258 | -0.242 | -0.002 | 0.111 | 0.051 | 0.011 |
| 4’-dimethylamino | -0.242 | -0.258 | -0.278 | 0.094 | 0.111 | -0.002 | 0.011 | 0.051 |
| 1-dimethylamino-2-nitro | 0.025 | -0.07 | -0.192 | -0.216 | -0.014 | 0.087 | 0.025 | 0.063 |
| 3’-dimethylamino-4’-nitro | 0.176 | 0.039 | 0.037 | -0.082 | 0.051 | 0.039 | -0.024 | 0.077 |
| 1,2- dimethylamino | 0.045 | 0.029 | -0.259 | -0.243 | -0.067 | 0.049 | 0.045 | 0.029 |
| 3’,4’-nitro | -0.169 | -0.165 | -0.002 | -0.108 | 0.081 | 0.009 | -0.169 | -0.165 |
| 1-nitro | -0.028 | -0.230 | -0.200 | -0.210 | 0.116 | 0.013 | -0.028 | -0.230 |
| 1’-nitro | -0.040 | -0.209 | -0.237 | -0.170 | 0.035 | 0.100 | -0.040 | -0.209 |
| 1-nitro-4-dimethylamino | -0.057 | -0.208 | -0.226 | -0.043 | 0.130 | 0.067 | -0.057 | -0.208 |
| 1’-dimethylamino-4’-nitro | -0.189 | -0.226 | -0.208 | -0.057 | 0.067 | 0.130 | -0.189 | -0.226 |
| 1-dimethylamino | 0.096 | -0.280 | -0.278 | -0.251 | 0.008 | 0.102 | 0.063 | -0.022 |
| 4’-nitro | -0.180 | -0.233 | -0.207 | -0.096 | 0.106 | 0.039 | 0.038 | 0.011 |
| 1-dimethylamino | 0.052 | -0.244 | -0.267 | -0.277 | -0.038 | 0.110 | 0.011 | -0.051 |
| 1’-nitro | -0.013 | -0.194 | -0.214 | -0.235 | 0.124 | 0.028 | 0.007 | -0.025 |
| 1-dimethylamino-4-nitro | 0.072 | -0.229 | -0.252 | -0.063 | -0.046 | 0.118 | 0.044 | 0.039 |
| 1’-dimethylamino-4’-nitro | 0.088 | -0.265 | -0.184 | -0.096 | 0.012 | 0.025 | -0.016 | -0.028 |

| [3.3]paracyclophane | 1 | 2 | 3 | 4 | 5 | 6 | 7 | 8 | 9 |
| --- | --- | --- | --- | --- | --- | --- | --- | --- | --- |
|  | 1’ | 2’ | 3’ | 4’ | 5' | 6' | 10 | 11 | 12 |
| [3.3]paracyclophane | -0.242 | -0.229 | -0.232 | -0.242 | -0.004 | -0.032 | 0.223 | 0.166 | 0.227 |
|  | -0.242 | -0.230 | -0.232 | -0.245 | -0.037 | -0.005 | 0.222 | 0.167 | 0.221 |
| 1-nitro | -0.039 | -0.205 | -0.242 | -0.204 | -0.035 | -0.015 | 0.221 | 0.159 | 0.219 |
|  | -0.219 | -0.234 | -0.238 | -0.226 | -0.004 | -0.033 | 0.220 | 0.166 | 0.218 |
| 1-dimethylamino | 0.075 | -0.236 | -0.271 | -0.249 | -0.022 | -0.019 | 0.218 | 0.162 | 0.225 |
|  | -0.206 | -0.219 | -0.254 | -0.24 | -0.01 | -0.02 | 0.223 | 0.169 | 0.238 |
| 1-dimethylamino | 0.108 | -0.201 | -0.273 | -283 | -0.027 | 0.005 | 0.203 | 0.155 | 0.194 |
| 1’-dimethylamino | 0.102 | -0.191 | -0.241 | -0.274 | 0.008 | -0.021 | 0.218 | 0.166 | 0.229 |
| 1-dimethylamino | 0.091 | -0.223 | -0.241 | -0.247 | -0.004 | -0.04 | 0.206 | 0.163 | 0.223 |
| 4’-dimethylamino | -0.247 | -0.224 | -0.223 | 0.091 | -0.004 | -0.04 | 0.223 | 0.163 | 0.206 |
| 1-dimethylamino-2-nitro | 0.091 | -0.223 | -0.241 | -0.247 | -0.004 | -0.04 | 0.206 | 0.163 | 0.223 |
| 3’-dimethylamino-4’-nitro | -0.247 | -0.224 | -0.223 | 0.091 | -0.004 | -0.04 | 0.223 | 0.163 | 0.206 |
| 1,2- dimethylamino | 0.066 | 0.062 | -0.236 | -0.232 | -0.017 | -0.043 | 0.196 | 0.161 | 0.177 |
| 3’,4’-nitro | -0.177 | -0.018 | -0.079 | -0.022 | -0.011 | 0.016 | 0.188 | 0.154 | 0.187 |
| 1-nitro | -0.036 | -0.194 | -0.218 | -0.224 | 0.023 | -0.054 | 0.175 | 0.166 | 0.225 |
| 1’-nitro | -0.049 | -0.198 | -0.232 | -0.224 | -0.044 | 0.000 | 0.233 | 0.162 | 0.222 |
| 1-nitro-4-dimethylamino | -0.019 | -0.195 | -0.261 | 0.066 | -0.018 | -0.027 | 0.240 | 0.154 | 0.193 |
| 1’-dimethylamino-4’-nitro | 0.083 | -0.255 | -0.211 | -0.034 | -0.044 | -0.041 | 0.173 | 0.174 | 0.246 |
| 1-dimethylamino | 0.096 | -0.211 | -0.262 | -0.269 | -0.032 | 0.002 | 0.195 | 0.159 | 0.231 |
| 4’-nitro | -0.196 | -0.211 | -0.227 | -0.076 | -0.015 | -0.026 | 0.213 | 0.172 | 0.229 |
| 1-dimethylamino | 0.104 | -0.193 | -0.275 | -0.275 | 0.005 | -0.007 | 0.185 | 0.168 | 0.150 |
| 1’-nitro | -0.006 | -0.221 | -0.217 | -0.213 | 0.300 | -0.048 | 0.201 | 0.169 | 0.249 |
| 1-dimethylamino-4-nitro | 0.083 | -0.218 | -0.235 | -0.056 | -0.023 | 0.013 | 0.184 | 0.16 | 0.205 |
| 1’-dimethylamino-4’-nitro | 0.092 | -0.177 | -0.211 | -0.070 | -0.056 | -0.067 | 0.181 | 0.168 | 0.227 |


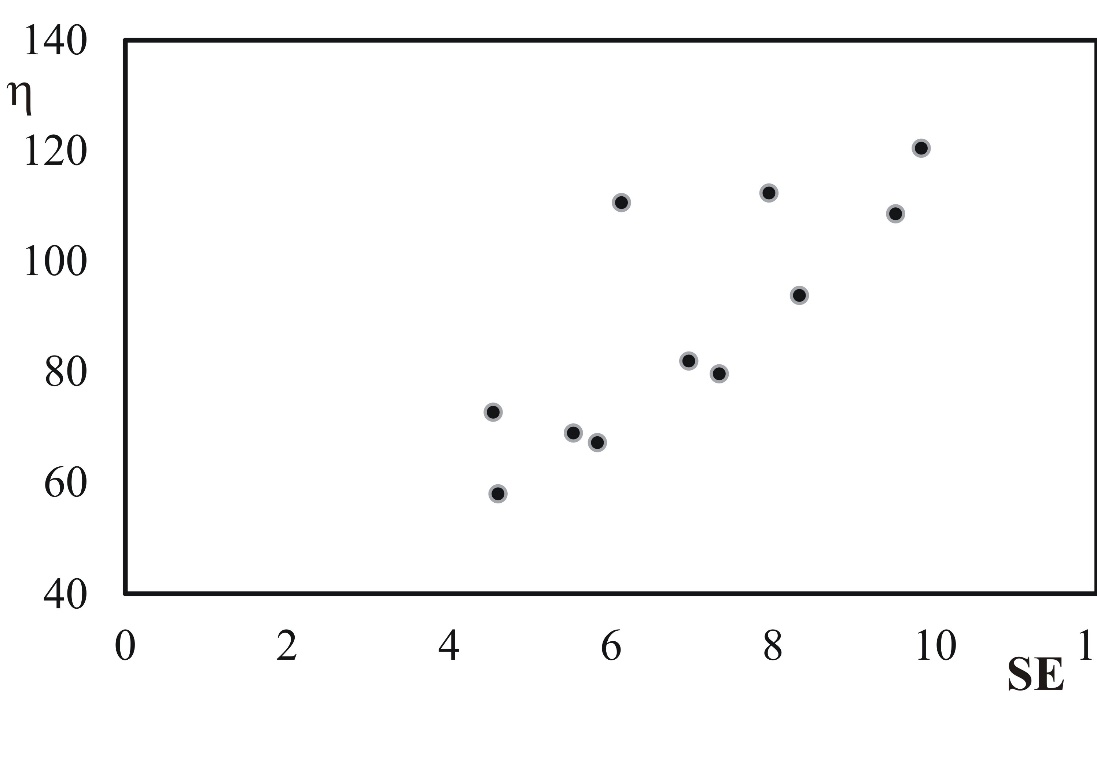


Fig S1. Values η for the different AIE values.


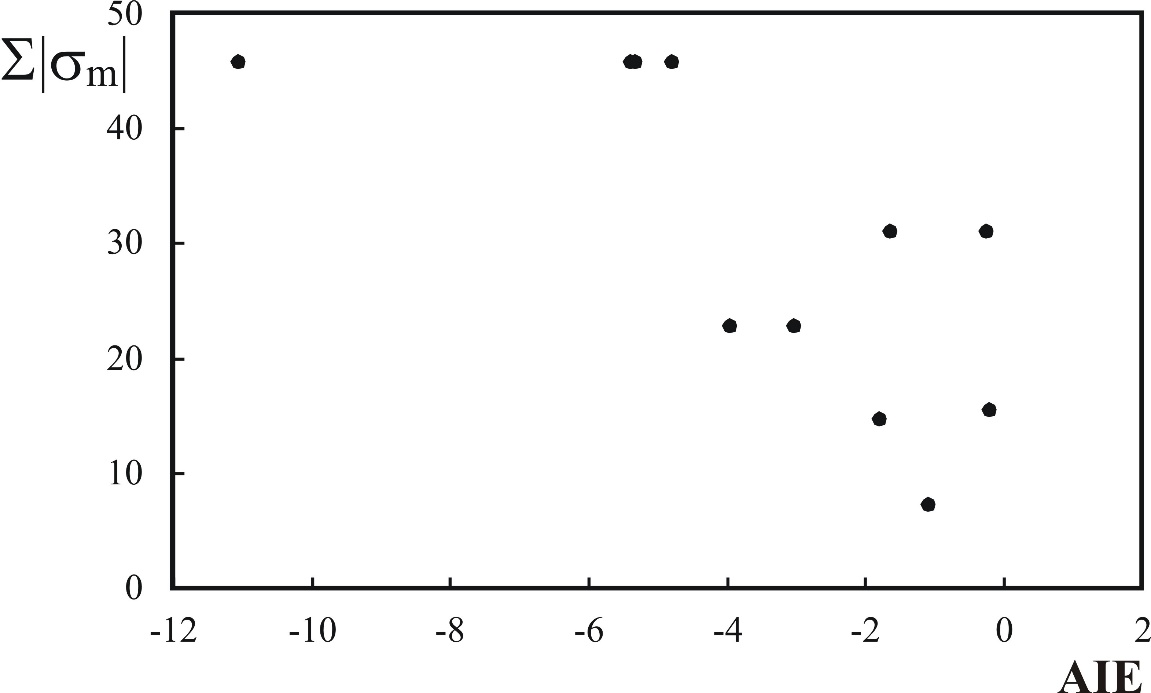


Fig. S2. Values Σ|σ_m_| for the different AIE values.


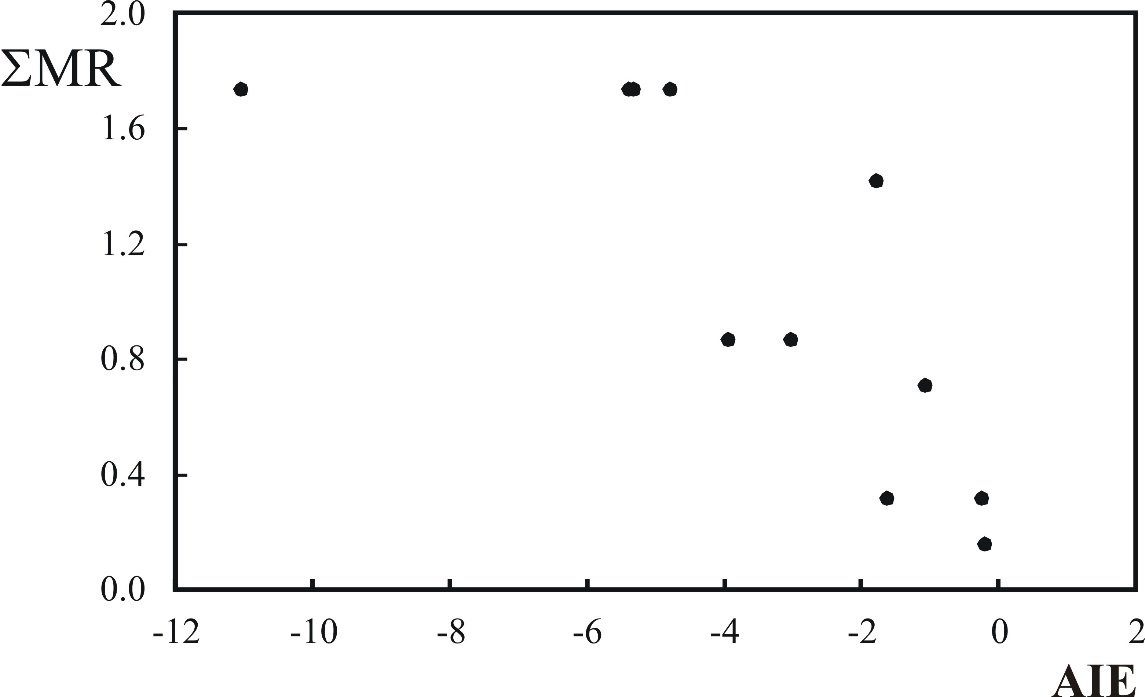


Fig. S3. Values ΣMR for the different AIE values.
